# Verteporfin selectively kills hypoxic glioma cells through iron-binding and increased production of reactive oxygen species

**DOI:** 10.1038/s41598-018-32727-1

**Published:** 2018-09-25

**Authors:** Katherine L. Eales, Edward A. Wilkinson, Garth Cruickshank, James H. R. Tucker, Daniel A. Tennant

**Affiliations:** 1 Institute of Metabolism and Systems Research, University of Birmingham, Edgbaston, Birmingham, B15 2TT UK; 2 School of Chemistry, University of Birmingham, Edgbaston, Birmingham, B15 2TT UK; 3 Department of Neurosurgery, University Hospitals Birmingham, NHS Foundation Trust, Birmingham, UK

## Abstract

Gliomas are highly malignant brain tumours characterised by extensive areas of poor perfusion which subsequently leads to hypoxia and reduced survival. Therapies that address the hypoxic microenvironment are likely to significantly improve patient outcomes. Verteporfin, a benzoporphyrin-like drug, has been suggested to target the Yes-associated protein (YAP). Increased YAP expression and transcriptional activity has been proposed in other tumour types to promote malignant cell survival and thus YAP-inhibitor, verteporfin, may be predicted to impact glioma cell growth and viability. Due to the extensive hypoxic nature of gliomas, we investigated the effect of hypoxia on YAP expression and found that YAP transcription is increased under these conditions. Treatment of both primary and immortalised glioblastoma cell lines with verteporfin resulted in a significant decrease in viability but strikingly only under hypoxic conditions (1% O_2_). We discovered that cell death occurs through a YAP-independent mechanism, predominately involving binding of free iron and likely through redox cycling, contributes to production of reactive oxygen species. This results in disruption of normal cellular processes and death in cells already under oxidative stress – such as those in hypoxia. We suggest that through repurposing verteporfin, it represents a novel means of treating highly therapy-resistant, hypoxic cells in glioma.

## Introduction

Gliomas are an extremely aggressive and heterogeneous group of primary brain tumour, accounting for over 80% of diagnosed malignant neoplasms of the brain and central nervous system^1^. Thought to be of neuroepithelial origin, gliomas are histopathologically categorised into ependymomas, astrocytomas or oligodendrogliomas, as outlined by the World Health Organisation (WHO)^2^. These tumours are then graded I-IV corresponding to the degree of malignancy exhibited, which can inform the clinical pathway^3^. Glioblastoma (GBM), a grade IV astrocytoma, is the most common and aggressive form of the disease with an abysmal five-year survival rate of around 5%^4,5^. Patients with GBM who undergo extensive surgical resection have a median survival of 4.2 months, which is only extended to 14.6 months upon the use of multimodal treatments such as chemoradiation therapy^5,6^. Discovering effective treatments for gliomas remains a significant challenge for researchers due to the extensive invasiveness of these tumours into the surrounding brain parenchyma^7^. Furthermore, gliomas are often very hypoxic tumours due to both their rapid growth rate and the presence of oedema^8^. This instigates further challenges in the design of therapeutics as this highly hypoxic subset of cells within these tumours often confer a high degree of drug resistance^8,9^. Despite significant efforts, treatments have remained largely stagnant since the development of Temozolomide in the 1990’s, which remains first-line therapy^10^. Surgical and technological developments have provided improvement in patient survival, but further significant improvements are needed. It is therefore imperative that we investigate into potential new pathways underlying glioma pathogenesis in the hope to discover novel and effective therapeutics.

One signalling pathway that has stimulated interest in the search for new glioma therapies is the Hippo pathway^11^. First elucidated from *Drosophila* genetic mosaic screens, this highly conserved pathway has emerged to regulate cellular processes underpinning tissue homeostasis and cell proliferation and differentiation^12–14^. Hippo pathway activity is dependent on the function of the transcriptional co-activators, the Yes-associated protein (YAP) and its close paralog transcriptional coactivator with PDZ-binding motif, TAZ (also known as WWTR1), which are downstream targets of a core kinase cascade comprised of mammalian Ste20-like kinases MST1/2 and large tumour suppressor LATS1/2^15^. Cellular localisation is critical to the function of YAP, with pathway inhibition allowing unrestricted translocation of YAP to the nucleus therefore permitting YAP to bind with various transcription factors such as tumour proteins p63/p73, runt-related transcription factor 1/2 (Runx1/2), octamer-binding transcription factor 4 (OCT4) and the most favoured interaction, the TEA domain (TEAD) family^15–17^. Dependent on the binding partner, these interactions result in the transcription of various downstream target genes largely associated with cell survival and proliferation such as connective tissue growth factor (*CTGF)* and cysteine-rich angiogenic inducer 61 (*Cyr61)* but also in some instances apoptotic genes such as *BBC3* (encoding the BH3-domain protein, puma)^16–19^. Strict control of this pathway is therefore imperative to healthy tissue development and cell growth and it is therefore of no surprise that its dysregulation has been closely linked with tumourigenesis^20^. YAP/TAZ amplification and nuclear localisation has been noted in various cancers such as hepatocellular carcinoma^21,22^, colorectal cancer^23,24^, lung cancer^25^ and ovarian cancer^26^ and is linked to a worse prognosis, tumour de-differentiation and increased tumour malignancy. Hyper-activation of YAP in cancer cells has also been shown to induce chemoresistance as well as promoting invasion, migration, epithelial-mesenchymal transition and aberrant tumour stemness^17,22,27^. Now identified as a potent oncogene, YAP has recently been linked to glioma growth and progression, with nuclear expression highly prevalent in GBM^11,28^. Recent studies have also described YAP to promote invasion of glioma cells^29^, whereas knockdown of YAP expression *in vitro* significantly reduced GBM growth^28^. Little is known about the abnormal regulation of YAP however a few studies have recently investigated the role of hypoxia in controlling YAP/TAZ activation in various cancers^30–32^, and therefore must also be examined in gliomas due to their extensive hypoxic nature.

Disrupting abnormal YAP activity in cells is of significant interest in cancer research and in recent years pharmacological screens have identified a potent YAP inhibitor^33^. Verteporfin (trade name Visudyne), a second-generation Food and Drug Administration (FDA) approved photosensitiser has been shown, in addition to its photodynamic properties, to disrupt the YAP-TEAD interaction consequently disrupting cell proliferation and oncogenic capabilities^33,34^. Drug repurposing is now a huge area of interest, particularly in cancer research due to the incredibly low rate of less than 5% of novel cancer therapies passing through phase I of clinical trials to approval^35^. Focus has therefore shifted in therapeutic discovery towards finding new purposes for clinically established drugs. Successful examples over the past decade have included anti-nausea drug Thalidomide - derivatives of which are now used in the treatment of multiple myeloma^36^ - as well as clinical trials proposing the repurposing of the type 2 diabetic drug Metformin in the treatment of breast cancer patients^37^. Verteporfin is therefore a positive candidate for reprofiling as in addition to its role as a YAP inhibitor, it is used clinically to treat neovascular age-related macular degeneration (AMD)^38^ and has previously been shown *in vitro* to reduce cancer growth^33,39,40^. Further investigations into whether this drug could be a useful therapeutic in the treatment of gliomas must therefore be conducted.

In this present study, we investigated the effect of hypoxia on YAP expression and that of it target genes in established GBM cell lines as well as exploring the effect of verteporfin on glioma cell survival. Interestingly, we observed for the first time a significant loss of cell viability after exposure to verteporfin specific to hypoxic conditions. The mechanism of this hypoxic cell death was further investigated and discovered to be a stress induced response, which was YAP-independent. Furthermore, we discovered that verteporfin has the ability to bind free metals in the form of free iron; an unprecedented finding that sheds more light on the potential mechanism of verteporfin-induced cell death. We therefore propose the potential of repurposing a clinically-available and approved therapeutic to target a significant hypoxic population of highly resistant and aggressive cells in gliomas.

## Methods

### Cell Culture

Human glioblastoma cell lines, U87 and U343 were obtained from CLS Cell lines Service GmbH (Germany) and cultured using Dulbecco’s Modified Eagle Medium High Glucose with L-Glutamine (DMEM; Sigma-Aldrich, UK) supplemented with 10% Foetal Bovine Serum (FBS; Thermo Fisher Scientific, UK). For hypoxia experiments, cells were cultured at 21% O_2_ and then transferred to the Whitley H35 Hypoxystation (Don Whitley Scientific, UK) set to 1% or 0.3% O_2_ for the stated amount of time. Primary GBM cell lines, T1 and T2, were derived from two distinct tumour loci during a single surgical procedure in November 2014 from a 32 year old male diagnosed with glioblastoma at the Queen Elizabeth Hospital Birmingham (QEHB) NHS Foundation Trust. Full details of cell line generation are outlined in the supplementary methods. All patients provided full, informed, written consent in accordance the Declaration of Helsinki. The use of patient material was approved by the Human Biomaterials Resource Centre (HBRC) at the University of Birmingham (research project number 13–165) and was ethically approved; reference committee for ethical approval 15/NW/0079 (NRES Committee North West – Haydock). All methods were performed in accordance with the relevant guidelines and regulations.

All cell lines were subject to routine mycoplasma testing using the EZ-PCR Mycoplasma Test Kit (Biological Industries, CT, USA) to ensure no contamination.

### Chemicals

All reagents used in this investigation were from Sigma-Aldrich (UK) unless otherwise stated.

Where specified, treatments were made using 5 μM verteporfin (VP; Tocris Bioscience, UK), 5 μM protoporphyrin IX (PPIX), 20 μM Z-VAD-FMK, 10 μM ALLN (VWR International, UK),1 and 3 μM Eeyarestatin-1 (Eer1; Tocris Bioscience, UK), 3 mM 4-hydroxy-TEMPO (TEMPOL) and 15 μM cyclosporin A (CsA), 50 μM necrostatin-1 (Nec-1), 1 mM dimethyloxalylglycine (DMOG) which were all from Cayman Chemical (MI, USA). VP concentration was kept at 5 μM for all experiments unless stated differently. All experiments involving VP were conducted in the dark (by the use of aluminium foil).

### Western Blotting

Western blotting was carried out as previously described^41^. 10% SDS-PAGE gels were used throughout. Membranes were cut post-blocking to probe for multiple antibodies in parallel. Primary antibodies used were: anti-Actin (A4700), anti-Connective Tissue Growth Factor (CTGF; Cusabio, TX, USA, CSB-PA09429A0Rb), anti-HIF-1α (BD Transduction, UK; 610959), anti-phosphorylated YAP (S127; Cell Signalling, UK, #4911), anti-YAP1 (Abcam, UK; ab56701 and ab52771) and anti-WWTR1 (TAZ; Atlas Antibodies, Sweden, HPA007415). Secondary antibodies were anti-mouse IgG HRP-Linked Secondary (Cell Signalling, UK; #7076) and anti-rabbit IgG HRP-Linked Secondary (Cell Signalling, UK; #7074).

### Quantitative Real-Time PCR

RNA was extracted using the RNeasy Mini Kit in accordance with the manufacturer’s protocol (Qiagen, UK). 1 μg RNA was transcribed using the Reverse Transcription System kit (Promega, UK). qPCR was performed on AB 7500 Real Time PCR System using the TaqMan® gene expression master mix (Applied Biosystems, UK). The following probes were used: *BBC3* (HS00248075_m1), *CTGF* (HS01026927_g1), *CYR61* (HS00998500_g1) and *YAP1* (HS00902712_g1) (Thermo Fisher Scientific, UK). Gene expression levels were normalised to *ACTB* (Actin; HS01060665_g1). Relative expression levels were calculated using the 2^−ΔΔCT^ method and compared to the relevant 21% O_2_ control.

### Sulforhodamine B Assay

Cells were fixed by adding 20% (v/v) ice-cold trichloroacetic acid (TCA; final concentration 25%) at 4 °C for 30 min. Wells were washed with dH_2_O, left to air-dry and intracellular protein was stained with 0.4% (w/v) sulforhodamine B (SRB) in 1% acetic acid for 10 min at room temperature. Wells were washed with 1% acetic acid to reduce non-specific staining and dried, after which SRB was dissolved using 50 mM Tris/HCL pH 8.8. Absorbance was quantified at 495 nm on FLUOstar OMEGA microplate reader (BMG LabTech, UK). Values were corrected for background absorbance and normalised within in each experiment as stated in the figure legends.

### Spheroid Analysis

U87 and U343 cells were cultured on 0.5% agarose coated plates using the liquid-overlay method^42^. Spheroids were either left to form for 7 days before treatment with verteporfin for a further 7 days or were allowed to form in the presence of verteporfin for up to 48 h. Images were taken using Leica DFC290 HD camera (20x objective) and diameter was measured using Image J (NIH). Any aggregated spheroids were excluded from analysis.

### Immunocytochemistry

Following treatment, cells were fixed with 4% paraformaldehyde (PFA; 15 min), quenched with 0.1 M glycine-PBS (5 min) and permeablised using 0.1% Triton-X-100 (5 min). Cells were then incubated with the following primary antibodies: anti-Calreticulin (Cell Signalling, UK; #12238), anti-YAP1 (Abcam, UK; ab56701 or ab52771), anti-8OHdG (Abcam, UK; ab62623), anti-Hoechst 33342 (NucBlue^®^ fixed cell stain ready probes™, Life Technologies, UK; R37606). Secondary antibodies Alexa fluor^®^ 488 goat anti-mouse IgG (H + L) and Alexa fluor® 488 goat anti-rabbit IgG (H + L) (Thermo Fisher Scientific, UK; A-11001 and A-11034 respectively) and DyLight 649 horse anti-mouse IgG (H + L) and DyLight 649 horse anti-rabbit IgG (H + L) (Vector Laboratories, UK; DI-2649 and DI-1649 respectively) were used. Cells were imaged on a ZEISS LSM 780 confocal microscope (ZEISS Microscopy, UK) using the Plan-Apochromat 100x/1.4 Oil DIC objective (1024 × 1024 pixels; 8-bit. Pinhole 100 µm). Pseudo-colouring was applied to the images using Zen 2012 SP1 software (ZEISS; black edition, version 8.1): UV channel (blue), 488 channel (green) and 633 channel (red).

### siRNA Knockdown and Plasmid Over-expression

Knockdown of YAP1 and TAZ was achieved using ON-TARGETplus SMARTpool human siRNA according to the manufacturer’s instructions (Dharmacon, UK). ON-TARGETplus SMARTpools used were non-targeting (D-001810-10), YAP1 (L-012200-00) and TAZ (siWWTR1; L-016083-00) at 25 nM using DharmaFECT 1 transfection reagent (Dharmacon, UK) 24 h post-seeding. Cells were analysed 72 h post-transfection (see figures for details). Overexpression of a triple mutant form of HIF-1α (HIF1αTM)^43^, which confers HIF-1α stabilisation under normoxic conditions, was achieved by transfecting cells 24 h post seeding with 3 μg of plasmid using DharmaFECT kb DNA transfection reagent (Dharmacon, UK) according to manufacturer’s protocol. Cells were analysed 28 h post-transfection.

### Flow Cytometry

After treatment, the cell media was collected separately for each condition. Cells were PBS washed, trypsinised (Gibco, UK) and added to the appropriate collected media. Cell suspensions were then left for 20 min. Cells were centrifuged (230 × g, 5 min), washed in PBS, centrifuged again and the pellet resuspended in 1x Annexin V binding buffer (BD Biosciences, UK). Cells were then stained with FITC Annexin V (BD Biosciences, UK) and propidium iodide (Life Technologies, UK) for 10 min at room temperature. Hydrogen peroxide (H_2_O_2_) was used as a positive control. Cells were analysed on the BD LSRFortessa X-20 cell analyser (BD Biosciences, UK) using BD FACSDiva Software. 10,000 cells were analysed per sample and data was processed using FlowJo software (version 8.7.3, FlowJo, LLC, USA).

### DCFDA Assay

Cells were treated with VP (2 h) followed by overlay of 2 × 2′,7′-Dichlorofluorescin diacetate (DCFDA; 20 µM final concentration) for the last 45 min of treatment. Wells were then washed with PBS and measured at 485 nm using the FLUOstar OMEGA microplate reader. To investigate whether VP-induced ROS production could be reduced, cells were co-treated with VP and free radical scavenger, 4-hydroxy-TEMPO. H_2_O_2_ was used as a positive control. Fluorescence values were corrected to the untreated cells for each experiment.

### Binding Assays

To assess iron-binding, 100 μM of ferric chloride hexahydrate (Fe^3+^) was added to 30 μM VP diluted in methanol. After one minute, the absorbance spectra were measured between 300–800 nm using the FLUOstar OMEGA microplate reader (BMG LabTech, UK). First order binding kinetic graphs were generated by calculating the ratio of absorbance at 670/686 nm when increasing concentrations (3–500 μM) of ferric chloride was added to VP (30 μM). Binding of Fe^3+^ was compared to increasing concentrations of Iron (II) chloride tetrahydrate (Fe^2+^; Honeywell Fluka, Romania), magnesium chloride (Mg^2+^; ThermoFisher Scientific, UK) and zinc chloride (Zn^2+^; Alfa Aesar, UK). A specific binding nonlinear line of best fit was drawn for each metal chloride.

### Statistical Analysis

Statistical analysis was performed using GraphPad Prism v7 (GraphPad Software, La Jolla California, USA). Data is presented as mean ± standard error of the mean (S.E.M). An unpaired T-test with Welch’s correction (2 samples), one-way ANOVA (>2 samples) or two-way ANOVA (>2 samples and oxygen conditions) with multiple comparisons post hoc tests (Bonferroni and Tukey) were conducted as appropriate. A Shapiro-Wilk normality test was conducted when appropriate. Statistical significance was assigned at *p < 0.05, **p < 0.01 and ***p < 0.001. Sample sizes and reproducibility are stated in the figure legends.

## Results

Glioblastomas are a highly therapy-resistant tumour type, with limited treatment options. One significant feature of these aggressive tumours is a high degree of severe hypoxia (<10 mmHg)^44^, which is a major determinant of treatment failure^45,46^. The transcriptional co-activator, YAP, has previously been described to regulate proliferation and apoptosis in a number of different tumour types, including GBM^11,20^. Additionally, YAP has been shown to translocate and promote transcription of its target genes in hypoxia^32^. Given that verteporfin, a YAP inhibitor, is already clinically-approved for the treatment of AMD, repurposing of this drug to treat GBM is an attractive option. However, there is currently no evidence to support a role for YAP in the survival of hypoxic glioblastoma.

### YAP and YAP-target gene expression are increased in glioma cells under hypoxia

**Figure 1:**
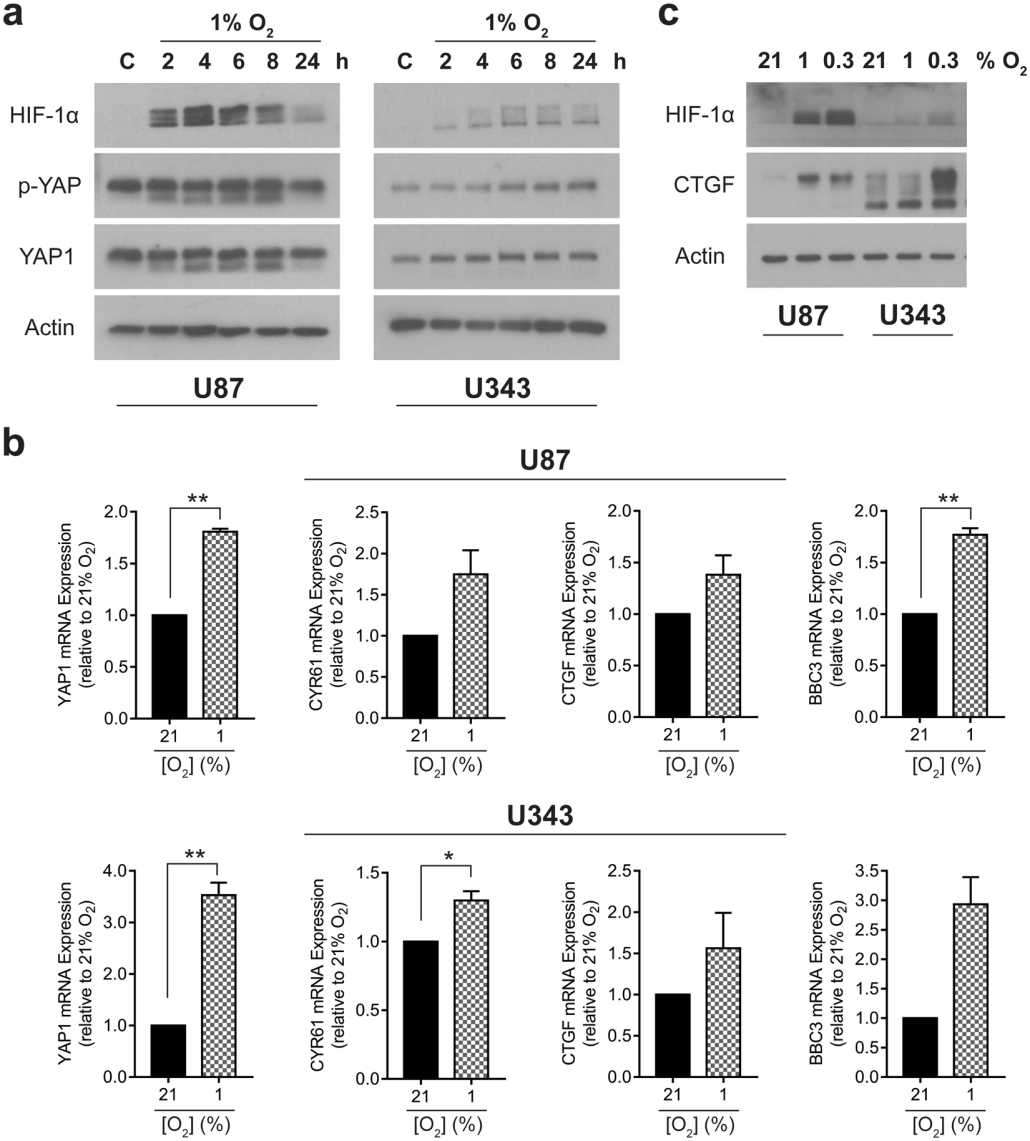
YAP1 transcription is upregulated in glioma cells under hypoxic conditions. **(a)** Western blots showing HIF-1α, phospho-YAP (p-YAP; S127), YAP1 and Actin expression in cells incubated under 21% O_2_ (C) for 24 h or 1% O_2_ for 2–24 h. (**b**) qRT-PCR analyses of YAP1 and YAP1-target gene expression after 8 h in 21% and 1% O_2_. (**c**) YAP1 target gene, CTGF protein expression (upper band) after incubation for 6 h at 21%, 1% and 0.3% O_2_. Blots are cropped for clarity from the same gel, delineated by white space. Full-length blots are presented in Supplementary Fig. S5. All experiments were conducted in biological triplicate, with qRT-PCR samples run in technical duplicate for each experiment. An unpaired T-test with Welch’s correction was conducted. Data is presented as mean ± S.E.M, *p < 0.05, **p < 0.01.

We examined the expression of YAP, and a subset of its target genes in two glioblastoma cell lines, U87 and U343, under hypoxia. We confirmed that incubation of these cells in 1% oxygen for up to 24 hours elicited a strong hypoxic transcriptional response in both cell lines, characterised by the increased expression of HIF-1α (Fig. 1a) and its target genes BNIP3, VEGFA and SLC2A1 (GLUT1; Supplementary Fig. S1). We also noted that over this time period, total YAP protein expression increased, although so did its inactivated phosphorylated form (Fig. 1a). Interestingly, in the U87 cells it appears that the hypoxia-induced increase may begin to decline at 24 hours, in contrast to that observed in the U343 cell line. As it was not clear from these data whether these changes were sufficient to induce transcription of YAP target genes, the expression of three such targets - CYR61, CTGF and BBC3 – as well as YAP were investigated at the mRNA level. We found that in both cell lines, YAP mRNA expression and expression of these reported target genes was increased (Fig. 1b). We also tested whether a lower oxygen tension would provoke a stronger YAP response, and observed that 0.3% oxygen resulted in at least as strong an upregulation of the target gene, CTGF, as 1% oxygen (Fig. 1c). Hence hypoxia leads to the increase in YAP gene expression as well as increase in downstream targets of YAP1 in glioma cells.

### Verteporfin selectively kills hypoxic cells via a YAP-independent mechanism

**Figure 2:**
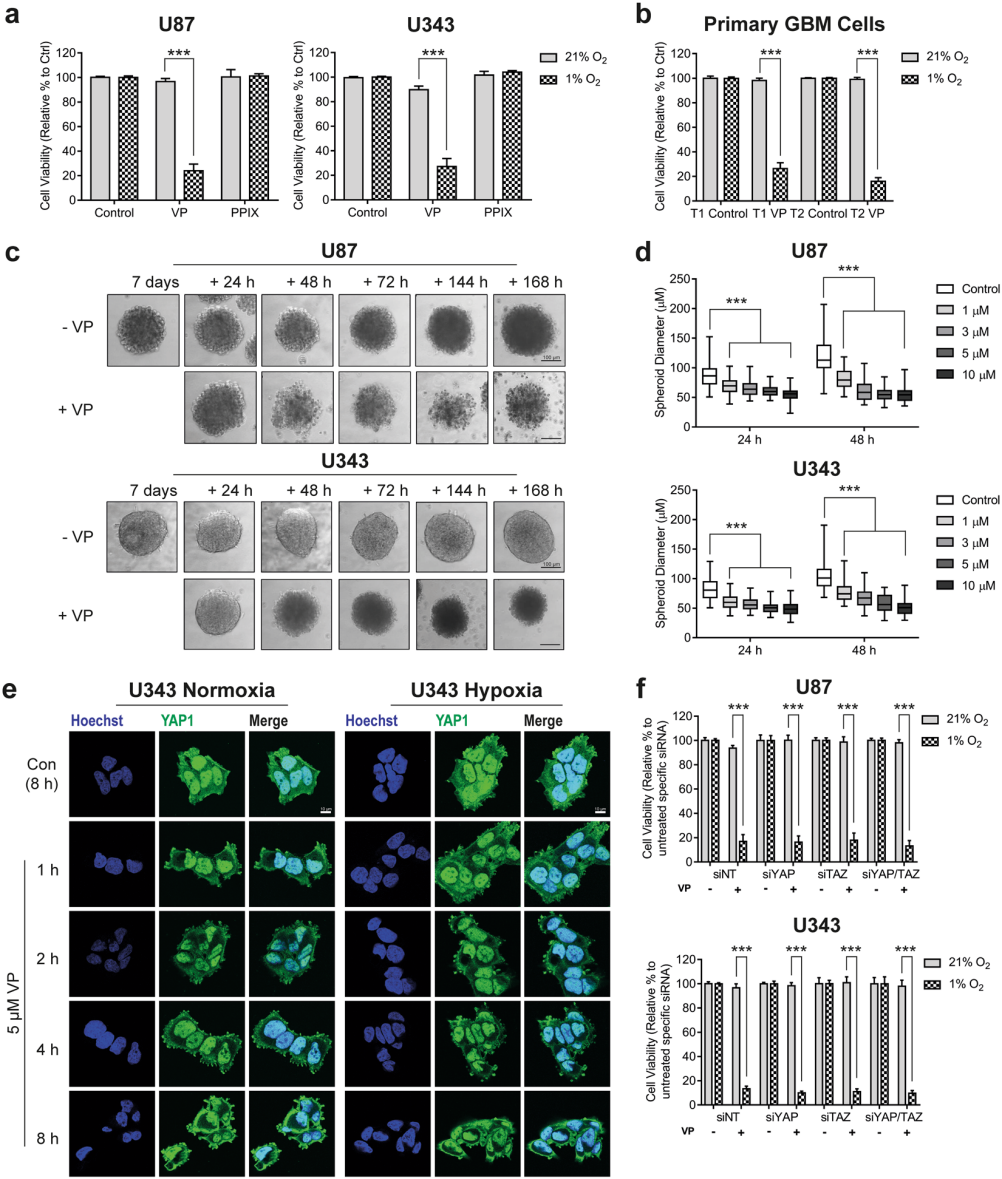
Verteporfin enhances cell death of glioma cells in a YAP-independent mechanism under hypoxic conditions. (**a**) Cell viability measured using SRB following 24 h treatment with VP (5 µM), and PPIX (5 µM) under 21% and 1% O_2._ (**b**) Viability of cells derived from a primary GBM tumour, T1 and T2, were also significantly reduced with 5 µM VP treatment but only in hypoxic (1% O_2_) conditions for 24 h. (**c**) Spheroids cultured for 7 days before treatment with vehicle (0.1% DMSO) or 5 µM VP for a further 7 days. Scale bar represents 100 µm. (**d**) Spheroids were grown for 24 and 48 h in the presence of vehicle (0.2% DMSO) or 1–10 µM VP and the average spheroid diameter recorded. Between 55–60 and 45–50 U87 spheroids and 50–55 and 35–40 U343 spheroids were analysed for each condition for both 24 and 48 h respectively. (**e**) Treatment of U343 cells with 5 µM VP between 1–8 h in both 21% and 1% O_2_ induced morphological changes. Scale bar represents 10 µm. (**f**) 5 µM VP for 24 h in 1% O_2_ caused a significant reduction in cell viability even after knockdown of YAP1 (siYAP), TAZ (siTAZ) and both YAP1 and TAZ (siYAP/TAZ) when compared to control (siNT). Viability is normalised to each untreated siRNA for each oxygen tension. All experiments were conducted in 3–4 independent experiments, with cell viability experiments run in technical triplicate for each independent experiment. A two-way ANOVA with Tukey post-hoc analysis was conducted. Cell viability data is presented as mean ± S.E.M, spheroid diameter data as box-and-whisker (min-max), ***p < 0.001.

An important facet of YAP activity is the suppression of apoptosis. We therefore wished to assess whether inhibition of YAP using the pharmacological agent, verteporfin, would remove the YAP-mediated protection from apoptosis in hypoxia. We incubated both U87 and U343 cells with verteporfin or another verteporfin-like compound - protoporphyrin IX (PPIX) which has also been proposed to inhibit YAP - in normoxia or hypoxia for 24 hours, and found that only verteporfin resulted in a significant hypoxia-specific decrease in cell viability (***p < 0.001; Fig. 2a and Supplementary Fig. S2). Furthermore, we found that the hypoxia-specific cell killing effect of verteporfin was also observed in two novel primary glioblastoma cell lines (Fig. 2b). In order to investigate whether verteporfin kills hypoxic cells within a more physiological *in vitro* model, we allowed both U87 and U343 to form spheroids. We found that verteporfin destabilised the spheroids over 7 days (Fig. 2c), and reduced diameter over 48 hours in a dose-dependent manner (Fig. 2d). Additionally treatment of spheroids during their formation inhibited their establishment (Supplementary Fig. S2).

We then investigated whether the killing effect of verteporfin could be observed as a result of a shorter, more clinically relevant treatment period. We therefore treated U87 and U343 cells with verteporfin for 4 hours, after which they were incubated without for the remaining 20 hours (Supplementary Fig. S2), and found that hypoxia-specific killing was also seen after this length of treatment. To visualise the effect of verteporfin on YAP localisation during the shorter treatment period, we stained for YAP at time points up to 8 hours, and found that YAP progressively re-localised near the plasma membrane (Fig. 2e and Supplementary Fig. S2). This result was unexpected, as we had hypothesised that verteporfin treatment would result in movement of YAP out of the nucleus into the cytoplasm where the inactive YAP form is localised. We therefore tested whether knockdown of YAP or the closely-related transcriptional regulator, TAZ, could phenocopy the hypoxia-specific killing by verteporfin. We confirmed successful knockdown of both proteins (Supplementary Fig. S2), and found that although siYAP, siTAZ or knockdown of both reduced cell number, knockdown was not sufficient to induce cell death in hypoxic cells (Supplementary Fig. S2). Interestingly, verteporfin still induced cell death in the absence of these proteins (Fig. 2f) therefore indicating that the hypoxia-specific killing of cells by verteporfin is YAP-independent.

### Stabilisation of HIF-1α in normoxia only partially sensitises cells to verteporfin

**Figure 3:**
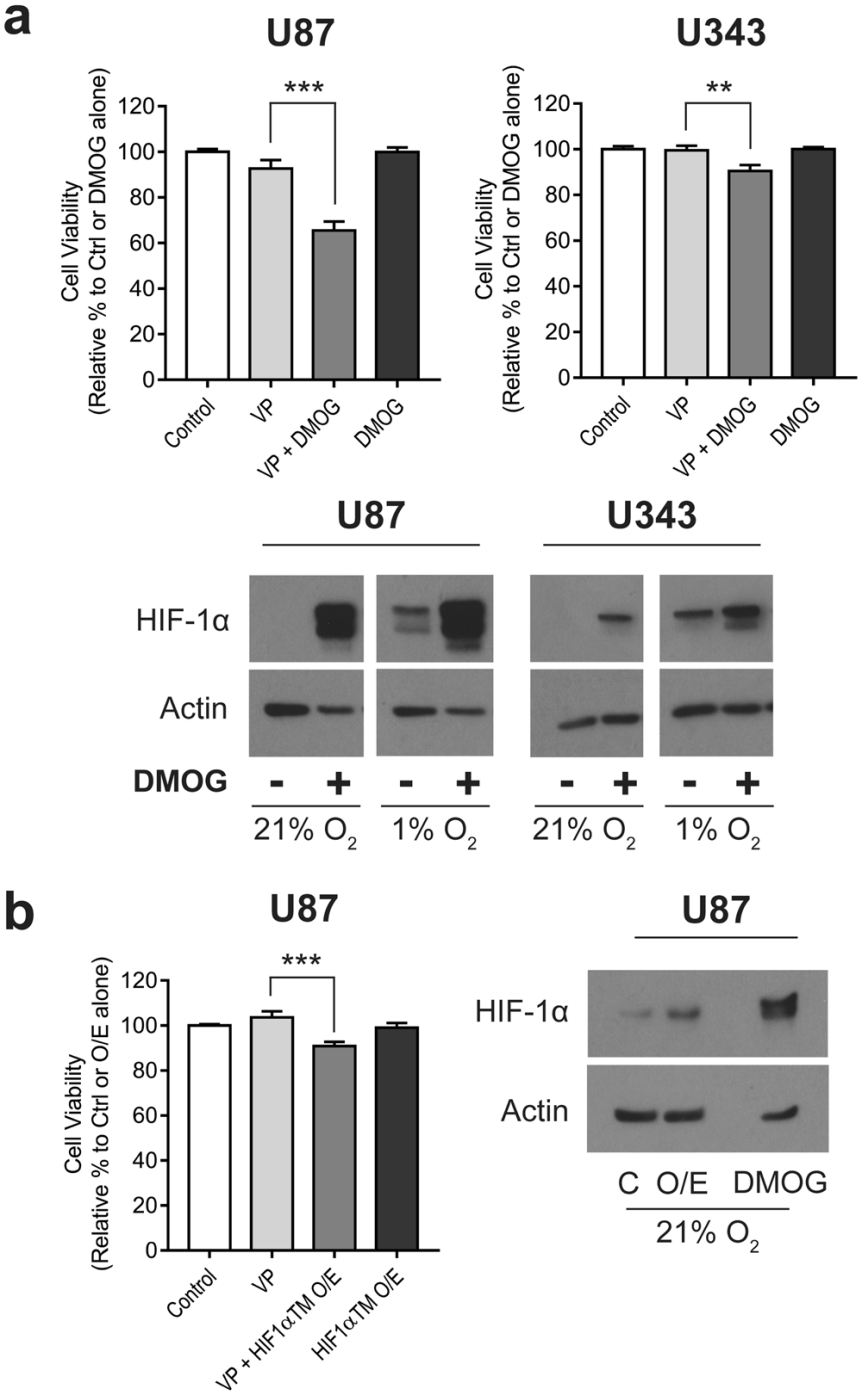
Hypoxic cell death with VP treatment is only partially dependent on HIF-1α. (**a**) Cells were treated with vehicle (0.1% DMSO), 5 μM VP, 5 μM VP + 1 mM DMOG or 1 mM DMOG alone. Overnight pre-treatment of DMOG (14–16 h) was conducted prior to VP addition for 4 h in 21% O_2_. Cell viability was normalised to control or untreated DMOG alone. Western blots show increased HIF-1α stabilization with DMOG treatment in 21% O_2_ and also under hypoxic conditions (1% O_2_). (**b**) A mutant form of HIF-1α (HIF1αTM) was over-expressed (O/E) in U87 cells to stabilize HIF-1α expression in normoxia. Cell viability was measured using SRB after 5 μM VP treatment for 4 h in 21% O_2_. Western blot shows increased stabilization of HIF-1α with mutant over-expression which is compared to expression reached with 1 mM DMOG for 20 h. Blots are cropped for clarity from the same gel, delineated by white space. Full-length blots are presented in Supplementary Fig. S5. All experiments were conducted in biological triplicate, with each cell viability experiment run in at least technical duplicate. A one-way ANOVA with Tukey post-hoc analysis was conducted. Cell viability data is presented as mean ± S.E.M, **p < 0.01, ***p < 0.001.

Due to verteporfin-induced hypoxic cell death being independent of YAP, we therefore wanted to investigate the mechanism by which verteporfin killed hypoxic glioblastoma cells. In hypoxia, HIF-1α stabilisation and activity of the resulting transcription factor HIF1 promotes major phenotypic changes that may increase sensitisation to verteporfin^45^. To examine the role of HIF1 in verteporfin-mediated cell death, we pharmacologically stabilised HIF-1α in normoxic conditions using dimethyloxylylglycine (DMOG; Fig. 3a) before treating with verteporfin. We found that DMOG partially sensitised both U87 and U343 cells to verteporfin (Fig. 3a), although the sensitisation of U343 was smaller than that observed in U87 cells. As an alternative approach, we also over-expressed a mutant form of HIF-1α (HIF1αTM) that is stable in normoxic conditions in U87 cells (Fig. 3b). Consistent with the pharmacological stabilisation of HIF-1α, we observed that expression of HIF1αTM produced a small, but significant increase in susceptibility to verteporfin-mediated cell death (***p < 0.001). However, neither of these approaches completely phenocopied the verteporfin-mediated cell death observed in hypoxia, suggesting that another aspect of hypoxic biology was likely the major determinant for the hypoxic sensitisation to verteporfin.

### Verteporfin-induced hypoxic cell death is partially preventable with pharmacological inhibitors of cell death

**Figure 4:**
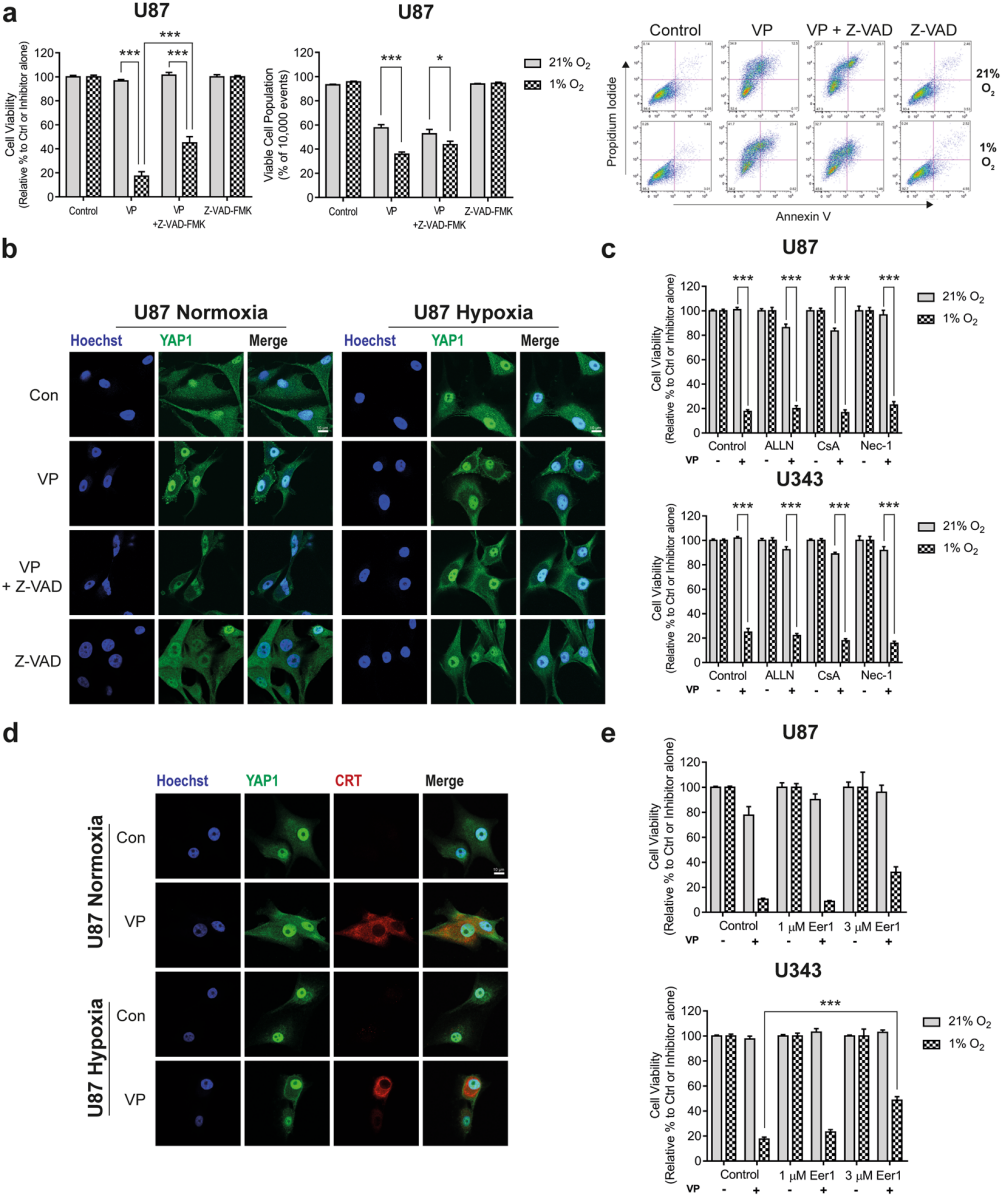
Verteporfin-induced cell death cannot be prevented by various cell death inhibitors and causes increased ER-stress. (**a**) Cell viability of U87 cells measured via SRB following 24 h treatment with vehicle (0.1% DMSO), 5 µM VP, 5 µM VP + 20 µM of pan-caspase inhibitor Z-VAD-FMK (Z-VAD; 1 h pre-treatment) or 20 µM Z-VAD-FMK alone in 21% and 1% O_2_. Cells were normalised to control or Z-VAD-FMK alone for each oxygen tension. U87 cell viability was also measured via flow cytometry after 8 h treatment with VP and Z-VAD-FMK in both 21% and 1% O_2_. Cells were dual stained with Annexin V and propidium iodide and the viable cell population was recorded for each condition. Representative flow plots are also presented for each condition. (**b**) U87 cells treated in 21% and 1% O_2_ for 8 h. The VP-induced morphological changes could not be rescued when co-incubated with the caspase inhibitor. (**c**) Cell viability was measured in the presence of other cell death inhibitors; 10 µM ALLN, 15 µM CsA and 50 µM Nec-1. Inhibitors were added 1 h prior to 5 µM VP treatment for 24 h. Cell viability was normalized to the either the control or the relevant untreated inhibitor for each oxygen tension. (**d**) Calreticulin (CRT) significantly increased in VP-treated (5 µM, 2 h) U87 cells in both 21% and 1% O_2_ indicating likely ER stress. (**e**) Eeyarestatin-1 (Eer1; 1 and 3 µM, 1 h pre-treatment) treatment partially rescued cell death after 5 µM VP treatment for 24 h in 1% O_2_. Viability is normalised to either control or untreated Eer1 for each oxygen tension. Experiments were conducted in 3–6 independent experiments, with SRB data being conducted in technical triplicate/quadruplicate for each biological experiment. Scale bar represents 10 µm. A two-way ANOVA with Bonferroni (Flow cytometry) or Tukey post-hoc analysis was conducted. Data is presented as mean ± S.E.M, *p < 0.05, ***p < 0.001.

In order to better define how verteporfin treatment resulted in hypoxia-specific cell death, we investigated whether inhibitors of different mechanisms of cell death could rescue the phenotype. We first assessed whether verteporfin induced caspase-dependent cell death, through the use of the caspase inhibitor, Z-VAD-FMK. We found that this inhibitor was only able to partially rescue the hypoxia-specific cell death (Fig. 4a,b and Supplementary Fig. S3) suggesting that this was not the major driver. We therefore investigated whether inhibitors of other forms of cell death could rescue viability and so treated cells with ALLN (calpain inhibitor), cyclosporin A (Ca^2+^-mediated cell death), necrostatin-1 (inhibitor of signalling pathways leading to necrotic cell death), pifithrin-α (PFT-α; inhibitor of p53 family of proteins) and chloroquine (CQ; inhibitor of autophagy). We observed however that none of these inhibitors were able to prevent verteporfin-induced cell death in hypoxia (Fig. 4c and Supplementary Fig. S3). We therefore examined whether verteporfin induced cell death through an endoplasmic reticular (ER) stress-mediated mechanism by staining of cells with the ER marker, calreticulin (CRT), which is upregulated in response to ER stress. We noted that CRT staining increased significantly after verteporfin treatment suggesting that ER stress may indeed be involved (Fig. 4d and Supplementary Fig. S3). However, treatment of cells with the inhibitor of the ER-associated degradation pathway, Eeyarestatin 1 (Eer1), which can inhibit cell death mediated as a result of ER stress, only partially reversed verteporfin-mediated cell death (Fig. 4e).

### Verteporfin increases production of reactive oxygen species and oxidative damage of DNA

**Figure 5:**
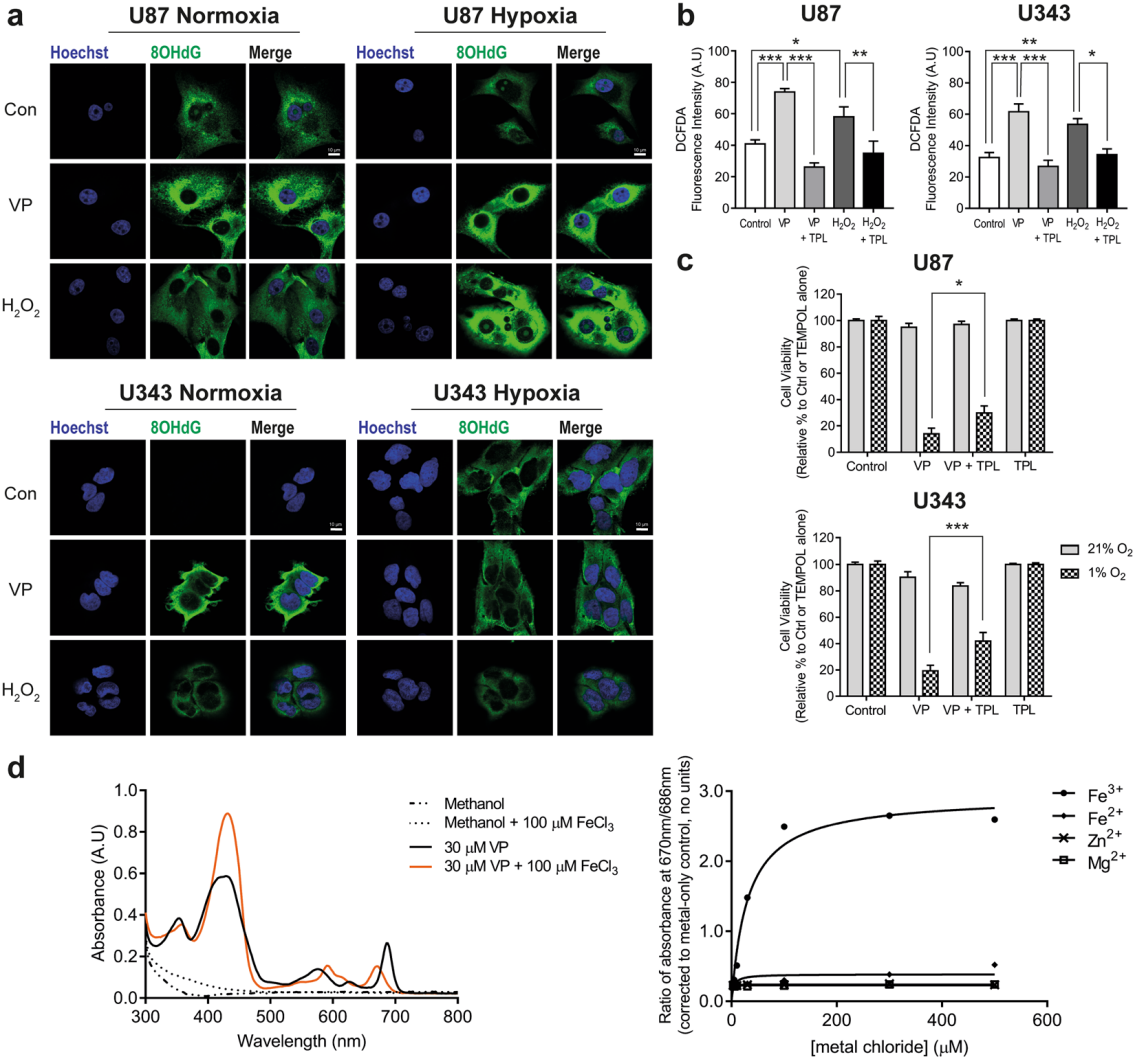
Verteporfin significantly induces reactive oxygen species in glioma cells and can bind free iron. (**a**) Treatment with 5 μM VP (2 h) caused a significant increase in DNA oxidation marker 8-Oxo-2′-deoxyguanosine (8OHdG). Hydrogen peroxide (H_2_O_2_; 100 μM) was used as a positive control. (**b**) A significant increase in cellular reactive oxygen species was detected using a DCFDA assay following treatment with 5 μM VP for 2 h in 21% O_2_. VP-induced ROS could be reversed if cells were co-treated with 3 mM TEMPOL (TPL; 2 h pre-treatment). H_2_O_2_ (50 μM) was used as a positive control. (**c**) Cell viability was measured using SRB following treatment with 5 μM VP for 24 h. Co-treatment with 3 mM TEMPOL was partially able to rescue cell viability in 1% O_2_. Cell viability was normalized to either the control or TEMPOL alone. (**d**) To assess whether VP could bind free iron, 100 μM of ferric chloride (Fe^3+^) was added to 30 μM VP and the absorbance spectra was measured between 300–800 nm. First order binding kinetic graphs were generated by calculating the ratio of absorbance at 670/686 nm when increasing concentrations (3–500 μM) of ferric chloride (Fe^3+^), ferrous chloride (Fe^2+^), zinc chloride (Zn^2+^) and magnesium chloride (Mg^2+^) was added to VP (30 μM). Experiments were conducted in 3–4 independent experiments, with data being conducted in a minimum of technical triplicate for each experiment. Scale bar represents 10 µm. A one-way ANOVA with Bonferroni (DCFDA data) or a two-way ANOVA with Tukey post-hoc analysis was conducted. Data is presented as mean ± S.E.M, *p < 0.05, **p < 0.01, ***p < 0.001.

Given that we had noted a significant degree of ER stress after verteporfin treatment, we also assessed the level of oxidative damage in normoxia and hypoxia, and found that it increased upon treatment in both conditions, but that in hypoxia the effect was more pronounced (Fig. 5a). Increased levels of reactive oxygen species (ROS) after verteporfin treatment was confirmed through DCFDA fluorescence – an effect that was reversed by treatment with the anti-oxidant, TEMPOL (Fig. 5b, and Supplementary Fig. S4). Hypoxia was also seen to significantly increase ROS production (*p < 0.05), which was exacerbated upon treatment with verteporfin when compared with normoxia (***p < 0.001; Supplementary Fig. S4). Indeed, we could also show that TEMPOL significantly reversed the hypoxia-specific cell death elicited by verteporfin (*p < 0.05, ***p < 0.001; Fig. 5c). Given that verteporfin is structurally similar to iron-binding porphyrins, we hypothesised that the mode of action was through binding of free iron resulting in a Fenton reaction. We therefore incubated verteporfin in the presence and absence of ferric iron (Fe^3+^) and assessed whether this altered the absorption profile of verteporfin in the UV/Vis spectrum (300–800 nm). We found that incubation with iron did indeed alter the profile, suggesting that iron was bound by the compound (Fig. 5d). We could also show that this shift is concentration dependent (Supplementary Fig. S4). When conducted under hypoxia, a similar binding profile was observed between ferric iron and VP indicating that binding was also possible under hypoxic conditions (Supplementary Fig. S4). In the case of both ferrous iron (Fe^2+^) and Zn^2+^, complexation was also indicated, albeit over a slower timescale, with no immediate changes to the spectrum upon addition of aliquots (Fig. 5d) but with considerable changes observed after 8 and 24 hours (Supplementary Fig. S4). In the case of Mg^2+^, no binding was observed even after incubation for 24 hours. These observations were supported by mass spectrometry data which indicated a 1:1 binding stoichiometry for the three complexes, with two pyrrole units deprotonated in each case. This data is consistent with the macrocycle acting as a tetradentate chelating ligand.

We therefore suggest that verteporfin mediates hypoxia-specific cell death in glioblastoma cells not through a YAP-dependent response, but rather through binding free iron and producing reactive oxygen species. Hypoxic cells demonstrate increased oxidative stress, and are likely susceptible to any interventions that further exacerbate this, providing rationale for the specificity of verteporfin. The ROS production induced by verteporfin leads to ER stress and DNA damage culminating in cell death.

## Discussion

Despite some improvements in the treatment of patients with glioma over the past 30 years, this tumour remains incurable. Treatments currently do not take account of some of the most significant aspects of glioma biology, which include widespread and severe hypoxia. It had previously been reported that the transcriptional co-activator, YAP, may play a role in hypoxic biology. We therefore investigated whether the YAP-inhibitor, verteporfin, could be repurposed to kill hypoxia glioma cells.

In agreement with previous studies^22,32^, our data suggested that hypoxia increased the expression of YAP, and upregulation of YAP target genes in two glioma cell lines (Fig. 1b,c). The hypoxic induction of YAP had also been suggested to play a role in proliferation and viability in these conditions, so we tested whether verteporfin could interfere with these roles, and found that it elicited a significant and specific loss of viability in both immortalised (Fig. 2a) and primary cell lines (Fig. 2b). We also found that verteporfin destabilised spheroid formation and proliferation dose-dependently (Fig. 2c,d, Supplementary Fig. S2), which has previously been observed in response to compounds that selectively target hypoxic cell survival^41^. Further investigation of the kinetics of the hypoxia-specific cell death observed suggested that this was a rapid process that was set in motion within the first hour of exposure (Fig. 2e). Given that changes in YAP-mediated gene expression would be expected to act over a longer time period, it was unlikely that the rapid induction of cell death in hypoxia was through YAP-specific inhibition by verteporfin. This finding is in agreement with a previous study demonstrating that verteporfin suppressed cell proliferation through a YAP-independent mechanism^47^.

Hypoxia results in a number of changes in phenotype – some through hypoxia-induced activity of the transcription factor, HIF-1, and some as a direct cause of reduced oxygen tension, such as changes in mitochondrial activity and ROS production. We therefore investigated whether DMOG, a reagent that stabilises HIF-1α in normoxia, as well as over-expression of a stable mutant of HIF-1α, could sensitise normoxic cells to verteporfin. We found that the stabilisation of the HIF-1 transcription factor by both methods resulted in a small but significant change in normoxic cell viability in response to verteporfin treatment (Fig. 3a,b), suggesting that the hypoxia-specificity of the effect was at least partially a result of HIF1-dependent changes in cell phenotype.

We therefore investigated the mode of cell death induced by verteporfin. Our initial studies using immunofluorescence had shown the presence of cytosolic vacuoles in response to verteporfin treatment (Fig. 2e and Supplementary Fig. S2), which suggested that the mode may not be through apoptosis – something we confirmed using the caspase inhibitor, Z-VAD-FMK, and Annexin V/PI staining (Fig. 4a,b, and Supplementary Fig. S3). However, we also found that inhibitors of other forms of cell death were not able to inhibit the action of verteporfin, with the exception of a small rescue by Eer1 (Fig. 4e). This mechanism was supported by calreticulin staining, showing enhanced ER stress induced by verteporfin, although this was not dependent on oxygen tension (Fig. 4d and Supplementary Fig. S3). We therefore hypothesise that the ER stress observed in hypoxia was likely above a threshold required to trigger cell death, while the normoxic stress was not. Interestingly, it has been previously shown that verteporfin results in an accumulation of protein aggregates^47,48^, which could be a result or a cause of the ER-stress we have described.

Our data had suggested that a specific property of verteporfin led to the hypoxia-specific death, compared to the closely related structure PPIX that did not induce the same effect (Fig. 2a and Supplementary Fig. S2). We demonstrate for the first time that verteporfin is capable of binding free iron (Fig. 5d and Supplementary Fig. S4), and therefore hypothesised that iron complexation may induce the production of ROS; production of which (by an undescribed mechanism) had previously been suggested to be a mode of action of verteporfin^48^, and importantly are known to be significantly increased by hypoxia. We were able to demonstrate that indeed verteporfin rapidly binds iron (Fig. 5d) in comparison with other physiologically relevant cations (Supplementary Fig. S4). This binding is likely to be central to the resulting production of ROS and oxidative stress (Fig. 5a,b, and Supplementary Fig. S4) through redox cycling, as previously described^49^. Treatment with the antioxidant was able to facilitate complete reversal of the increased ROS (Fig. 5b and Supplementary Fig. S4), and significantly, although not completely, reverse the effect of verteporfin on cell viability (Fig. 5c).

In the treatment of AMD, activity of the drug is modulated by exposure of the eye to red light, which increases ROS production. We have shown that this drug elicits ROS production in all conditions, even without the use of light. However, the ROS level is likely lower in these conditions, which likely results in hypoxia-specificity due to the increase oxidative stress observed in these conditions (Supplementary Fig. S4). Since verteporfin can induce these effects in the absence of light, it is therefore likely that additional interventions to deliver photodynamic therapy to the tumour would not be required. Low doses of verteporfin has been suggested to inhibit the growth of glioma cells without light treatment^50^, while higher concentrations appear to lead to a complete loss of viability^48^. These data alongside ours suggest that it will be important to accurately identify the appropriate therapeutic window, or whether methods such as local depots of the drug may be an optimal method for treatment in order to reduce the impact of light on drug activity. It is of interest that the successful treatment of AMD appears to be through the selective accumulation of verteporfin in areas of the retina that contain abnormal vessels^38^. This may suggest that cellular uptake of verteporfin is increased in hypoxic conditions, which may also contribute to the hypoxia-specific cell killing we observed.

In conclusion we have shown that verteporfin, an FDA-approved drug with a known toxicity profile, can specifically kill hypoxic glioma cells. Hypoxic cells are among the most therapy resistant cells in tumours, and therefore represent a significant clinical challenge. Therapies that can modulate this resistance, or directly kill hypoxic cells may significantly improve patient outcomes in a number of tumours types, including glioma.

## Electronic supplementary material

Supplementary Information

## Data Availability

The datasets used and analysed during the current study are available from the corresponding author on reasonable request.
