# Supplementary material for: Verteporfin selectively kills hypoxic glioma cells through iron-binding and increased production of reactive oxygen species
Source: Sci Rep. 2018 Sep 25;8:14358. doi: 10.1038/s41598-018-32727-1 (PMC6156578; doi:10.1038/s41598-018-32727-1)
Supplement: Supplementary file 1 — Supplementary Information [file 41598_2018_32727_MOESM1_ESM.pdf]

Supplementary Information for:

Verteporfin selectively kills hypoxic glioma cells through iron-binding and increased production of reactive oxygen species.

Katherine L Eales[1], Edward A Wilkinson[2], Garth Cruickshank[3], James H R Tucker[2] and Daniel A Tennant[1,*]

[1]Institute of Metabolism and Systems Research, University of Birmingham, Edgbaston, Birmingham. B15 2TT, UK

[2]School of Chemistry, University of Birmingham, Edgbaston, Birmingham. B15 2TT, UK

[3]Department of Neurosurgery, University Hospitals Birmingham, NHS foundation Trust, United Kingdom

[*]Corresponding author: Institute of Metabolism and Systems Research, University of Birmingham, Edgbaston, Birmingham. B15 2TT, UK d.tennant@bham.ac.uk

Contents:

1. Supplementary Methods

2. Supplementary Figures S1-5

Supplementary Methods

Quantitative Real-Time PCR of Hypoxia Genes

Quantitative RT-PCR was conducted as stated in the main paper. Hypoxia genes were quantified using the following probes: BNIP3 (HS00969291_m1), SLC2A1 (GLUT1; HS00892681_m1) and VEGF-A (HS00900055_m1) (Thermo Fisher Scientific, UK).

GBM Cell Line Generation

The patient had previously undergone a right temporal craniotomy in March 2014 followed by cranial radiotherapy over six weeks to 54GY in 30 fractions with continuous oral Temozolomide, followed by a course of 5/30 adjuvant Temozolomide in July 2014. Immediate 'post-op' imaging showed two distinct areas/ foci of residual tumour medial and lateral in the right temporal lobe (Supplementary Fig. S2). Follow-up MRI imaging in October 2014 showed that one area (labelled ‘A’; Supplementary Fig. S2, from which T1 was derived) had increased in size, while the other (labelled ‘B’; Supplementary Fig. S2, from which T2 was derived) was smaller/the same size than when previously imaged suggesting a therapeutic response in one but not the other (Supplementary Fig. S2). During reoperation in November 2014 samples were removed from sites ‘A’ and ‘B’ for derivation of cell lines – T1 (deemed more therapy-resistant due to its recurrent growth) and T2 (suggested more therapy-sensitive), respectively. Histopathological analysis of the tumour biopsy was conducted by the Pathology department at QEHB.

Primary cell lines T1 and T2 were generated by placing the tumour biopsy pieces into a sterile petri dish, washed three times with 1x PBS, and cut into small pieces using a sterile scalpel. The dissected pieces were washed again with 1x PBS, transferred to a 50 ml falcon tube in 1x PBS and centrifuged (230 x g, 5 min). The PBS was removed and the pieces subjected to a second round of washing and centrifugation (230 x g, 5 min). Following centrifugation, the pieces were trypsinised for 10 min and neutralised using DMEM High Glucose with L-Glutamine (Sigma-Aldrich, UK) supplemented with 10% FBS (Thermo Fisher

Scientific, UK). The pieces were again centrifuged (230 x g, 5 min), media removed and the tissue pellet resuspended in DMEM containing antibiotics (1% Penicillin/Streptomycin, 0.1% Gentamycin and 1% Amphotericin B; Sigma-Aldrich, UK). The pieces were then mechanically dissociated by repeated pipetting until a homogenous solution was achieved. This cell solution was then seeded onto flasks and cultured under standard conditions.

Cell Viability following VP Removal

Cells were treated with 5 µM verteporfin for either 4 h followed by removal and replacement with DMEM for 20 h or VP treated for 24 h. Following treatment, cell viability was quantified using the SRB method as previously stated.

Protoporphyrin IX and Verteporfin dose response

U87 and U343 cells were treated with vehicle (0.2% DMSO) or increasing concentrations of protoporphyrin IX or verteporfin (1-10 µM) for 24 h in 21% or 1% O_2. Following treatment, cell viability was quantified using the SRB method as previously stated.

Assessment of Cell Growth Following Gene Knockdown

To investigate the effect of YAP1 and TAZ on glioma cell growth, gene knockdown was achieved as stated in the main methods. Cell proliferation was assessed 72 h post transfection using the SRB method.

Treatment p53 and Autophagy Inhibitors

To investigate the mechanism of verteporfin-induced cell death, U87 and U343 cells were treated with 5 µM verteporfin following 1 h pre-treatment with either 30 µM of p53 inhibitor pifithrin-α hydrobromide (PFT-α; Tocris Bioscience, UK) or 10 µM of autophagy inhibitor chloroquine (CQ). Cells were left for 24 h in 21% and 1 % O2, followed by cell viability assessment using the SRB method as stated in the main paper.

DCFDA Assay

Reactive oxygen species were quantified using a DCFDA assay as described in the main methods. To assess whether PPIX was capable of inducing ROS, U343 cells were treated with vehicle (0.1% DMSO), 5 µM PPIX, or 5 µM PPIX + 3 mM TEMPOL for 2 h in 21% and 1% O_2 and processed as previously described. To investigate the effect of hypoxia on ROS production in the presence of VP, U343 cells were treated with vehicle (0.1% DMSO), 5 µM VP or 5 µM VP + 3 mM TEMPOL for 2 h in both 21% and 1% O_2 and again processed as outlined in the main methods.

Binding Assays

Increasing concentrations (3-500 µM) of ferric (Fe^{3+}) chloride hexahydrate were added to 30 µM verteporfin diluted in methanol. The absorbance was measured after one minute at 300-800 nm using the FLUOstar OMEGA microplate reader (BMG LabTech, UK). 100 µM Iron (II) chloride tetrahydrate (Fe^{2+}), magnesium chloride (Mg^{2+}) and zinc chloride (Zn^{2+}) were also added to 30 µM verteporfin diluted in methanol and the absorbance spectra read at 300-800 nm. Absorbance values were also recorded 8 and 24 h after addition of 3-500 µM of Fe^{3+}, Fe^{2+}, Mg^{2+} and Zn^{2+} to 30 µM verteporfin. First order binding kinetics graphs were generated by calculating the ratio of absorbance at 670/686 nm. For hypoxic studies, increasing concentrations (3-500 µM) of ferric (Fe^{3+}) chloride hexahydrate were left under 21% or 1% O_2 for 4 h before 30 µM VP was added and the absorbance spectra read at 300-800 nm. For the first order binding kinetics graph the ratio of absorbance values were calculated at 670/686 nm for normoxia however it was noted that the VP spectra peak at 686 nm had shifted to 688 nm and 670 nm had shifted to 672 nm and so for hypoxic conditions the ratio was calculated at 672/688 nm.

Mass Spectrometry

Ferric chloride hexahydrate (Fe^{3+}), Iron (II) chloride tetrahydrate (Fe^{2+}), magnesium chloride (Mg^{2+}) and zinc chloride (Zn^{2+}) was added in excess to verteporfin and dissolved using 99.8% methanol (extra-dry over molecular sieve; Thermo Fisher Scientific, UK). Samples

were then subject to low-resolution MALDI-TOF LD^+ mass spectrometry using the service provided by The Centre for Chemical and Materials Analysis in the School of Chemistry at the University of Birmingham.

Supplementary Figures:

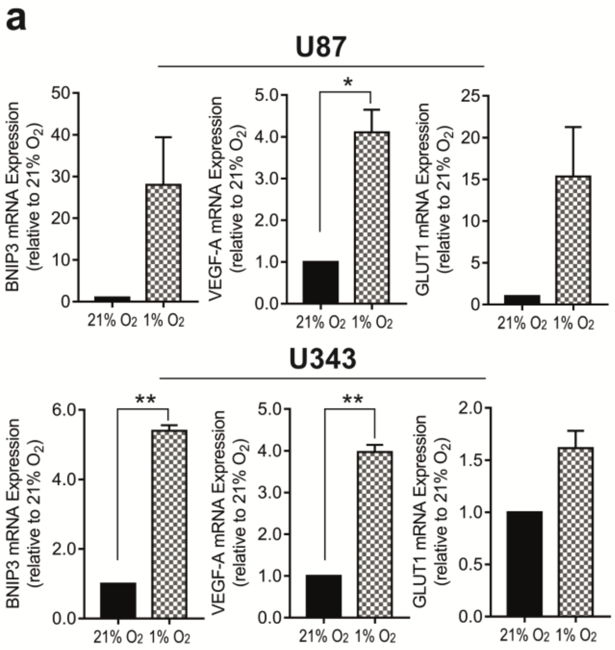

Supplementary Figure S1: HIF-1α target genes are increased in hypoxia. (**a**) qRT-PCR analyses of hypoxic gene expression, BNIP3, VEGF-A and GLUT1 in U87 and U343 cells under 1% O_2 for 8 h. Gene expression levels in each sample were normalised to ACTB (Actin) which was used as a housekeeping gene. Relative expression levels in hypoxia were normalised to the relevant 21% O_2 control for each sample. All experiments were conducted in biological triplicate, with each experiment run in technical duplicate. An unpaired T-test with Welch's correction was conducted. Data is presented as mean ± S.E.M, * $p<0.05$ ** $p<0.01$.

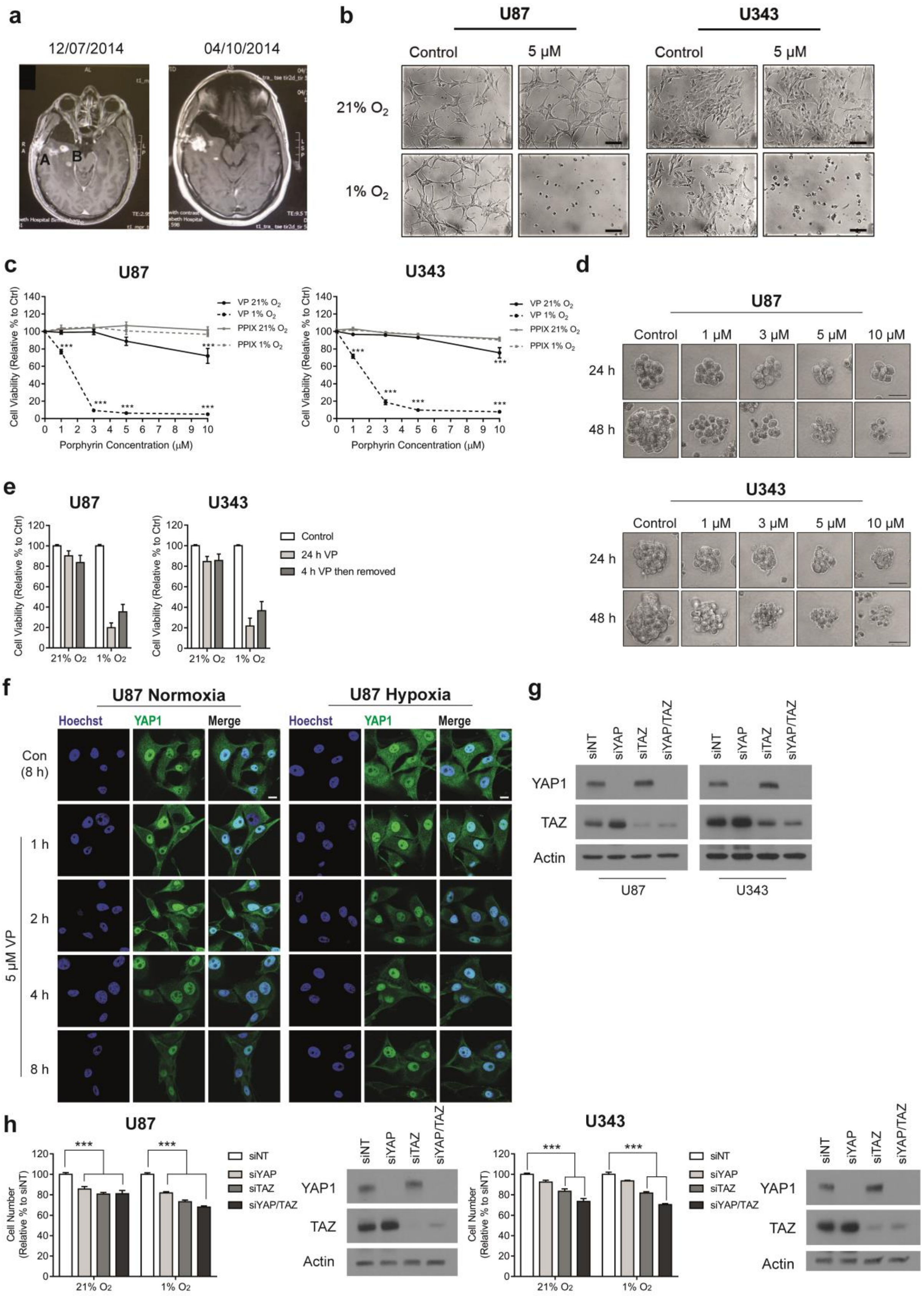

Supplementary Figure S2: **YAP-inhibitor verteporfin reduces spheroid growth and induces hypoxic cell death whereas YAP knockdown reduces cell number. (a)** MRI

scans from a 32 year old male diagnosed with glioblastoma taken at two different dates. Scans show progression of the main right temporal GBM therapy-resistant lesion (A) between 12/7/2014 and 04/10/2014. **(b)** Images of U87 and U343 cells following treatment with vehicle (0.1% DMSO) or 5 μM VP for 24 h in both 21% and 1% O_2. Upon VP exposure, cells remained viable under normoxic conditions but died under hypoxic conditions. Scale bar 100 µm. **(c)** Cell viability determined via SRB of U87 and U343 cells following treatment with increasing concentrations (1-10 µM) of verteporfin or protoporphyrin IX for 24 h in both 21% and 1% $O_{2.}$ Cells remained viable in normoxia, although exhibited reduced growth with 10 µM VP. Under hypoxia, only VP induced significant cell death. Treatment was normalised to the vehicle control for each oxygen tension. **(d)** U87 and U343 cells grown as spheroids in the presence of either vehicle (0.2% DMSO) or 1-10 μM VP for 24 and 48 h. Scale bar represents 50 µm. **(e)** Cell viability determined via SRB following treatment of either vehicle (0.1% DMSO) or 5 μM VP for 4 h after which VP was removed and cells incubated in media for 20 h or 5 μM VP for 24 h in both 21% and 1% O_2. Treatment was normalised to the vehicle control for each oxygen tension. **(f)** YAP1 plasma membrane localisation is increased in U87 cells treated with 5 μM VP for 1-8 h and is enhanced under hypoxic conditions (1% O_2). YAP1 appears to still be nuclear localised but its presence in the cytoplasm diminishes. Scale bar represents 10 µm. **(g)** Western blots showing considerable knockdown of YAP1, TAZ and both YAP1 and TAZ protein in samples corresponding to experiments run in Figure 2F. **(h)** Cell proliferation analysis of U87 and U343 cells after transfection with 25 nM siRNA against non-targeting (siNT), YAP1 (siYAP), TAZ (siTAZ) and YAP1 and TAZ together (siYAP/TAZ). Cells were transfected and cultured in 21% and 1 % O_2 for 72 h post-transfection after which cells were fixed for SRB analyses. Cell growth was normalised as a percentage of the control (siNT) for each oxygen tension. Western blots confirm knockdown of targets in both cell lines. Blots are cropped for clarity from the same gel, delineated by white space. Full length blots are presented in supplementary figure S5. Experiments were run in at least biological triplicate. A one-way (Fig. S2c) and two-way (Fig

S2h) ANOVA with Tukey post-hoc analysis was conducted. Data is presented as mean ± S.E.M. *** p<0.001.

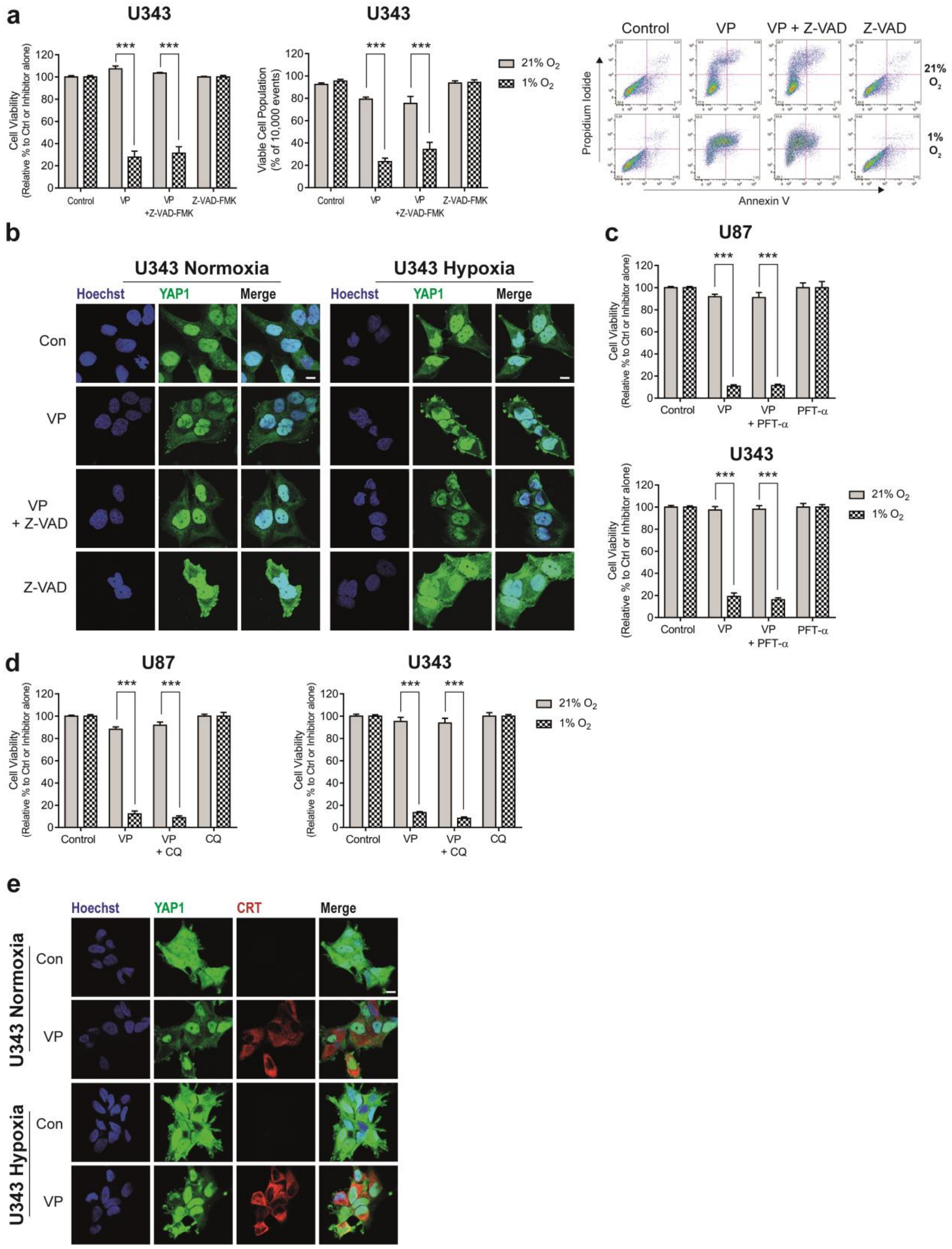

Supplementary Figure S3: Verteporfin-induced cell death cannot be prevented by various cell-death inhibitors and causes increased ER-stress. **(a)** U343 cells were treated with either vehicle (0.1% DMSO), 5 µM VP, 5 µM VP + 20 µM Z-VAD-FMK or 20 µM

Z-VAD-FMK alone. Cells were pre-treated for 1 h with Z-VAD-FMK prior to treatment with VP. Cells were incubated for 24 h in 21% and 1% O_2 after VP treatment and cell viability was measured through SRB. Cells were normalised to control or Z-VAD-FMK alone for each oxygen tension. Viability of U87 cells was also measured via flow cytometry after 8 h treatment with VP in both 21% and 1% O_2. Cells were dual stained with Annexin V and propidium iodide and the viable cell population was recorded for each condition. Representative flow plots are also presented for each condition. **(b)** U343 cells were treated with vehicle (0.1% DMSO; con), 5 µM VP, 5 µM VP + 20 µM of Z-VAD-FMK or 20 µM Z-VAD-FMK alone in 21% and 1% O_2 for 8 h. The morphological changes seen with VP treatment could not be rescued when co-incubated with the caspase inhibitor. **(c and d)** Glioma cells were treated with p53-inhibitor, pifithrin-α (PFT-α; 30 µM) and autophagy inhibitor chloroquine (CQ; 10 µM) and treated with 5 µM VP for 24 h. Cells were pre-treated with the inhibitors for 1 h prior to VP treatment. These inhibitors were not able to rescue VP-induced cell death in 1% O_2. Viability was normalised to either control or untreated inhibitor. **(e)** Calreticulin expression is significantly increased in U343 cells following exposure to 5 µM VP for 2 h in both 21% and 1% O_2. Experiments were conducted in at least biological triplicate with SRB data was conducted in a minimum of technical triplicate for each experiment. Scale bar represents 10 µm. A two-way ANOVA with Tukey post-hoc analysis was conducted. Data is presented as mean ± S.E.M, ***$p<0.001$.

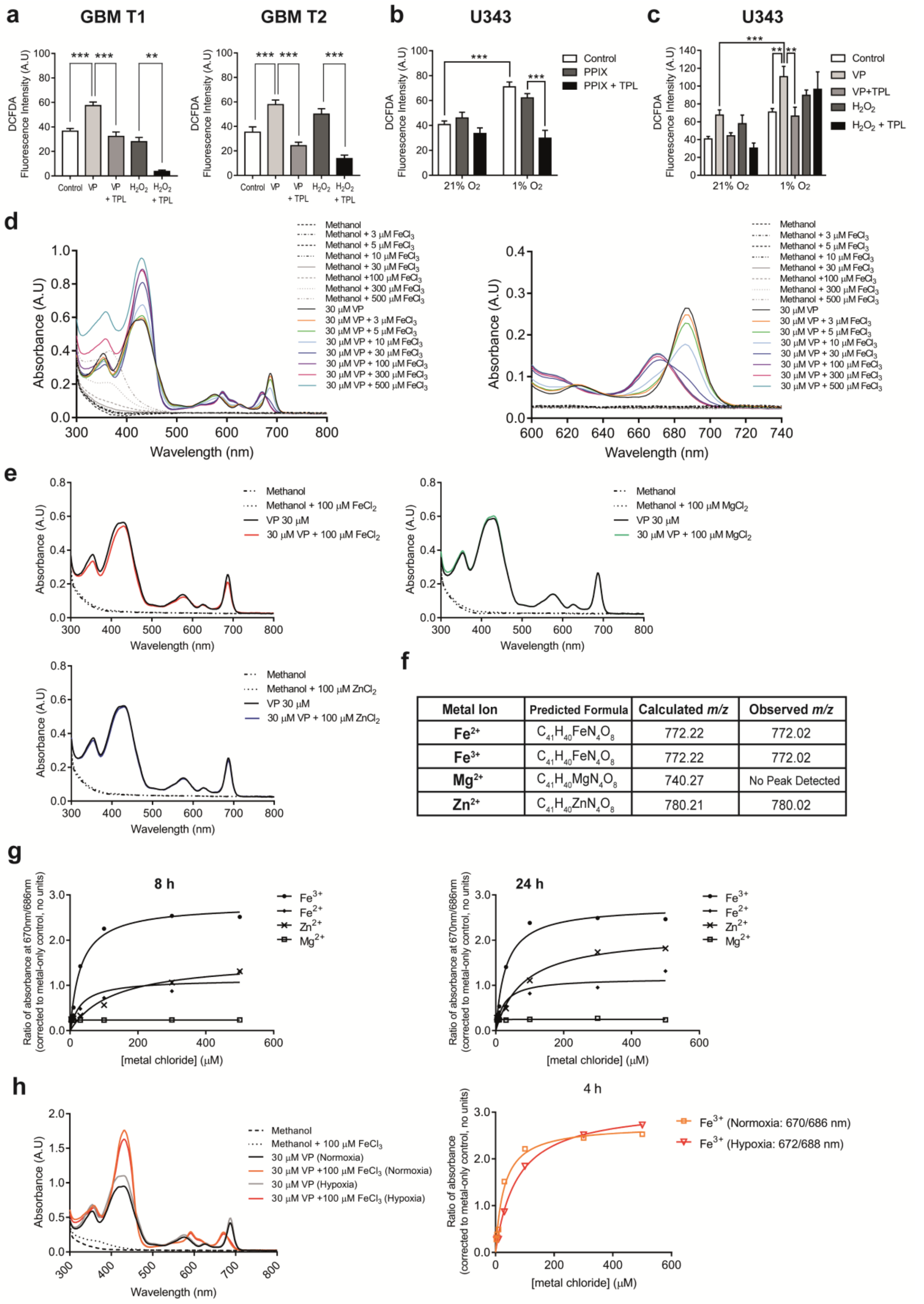

Metal Ion	Predicted Formula	Calculated *m/z*	Observed *m/z*
Fe^{2+}	$C_{41}H_{40}FeN_4O_8$	772.22	772.02
Fe^{3+}	$C_{41}H_{40}FeN_4O_8$	772.22	772.02
Mg^{2+}	$C_{41}H_{40}MgN_4O_8$	740.27	No Peak Detected
Zn^{2+}	$C_{41}H_{40}ZnN_4O_8$	780.21	780.02

Supplementary Figure S4: **Verteporfin increases reactive oxygen species and can avidly bind ferric iron**. **(a)** Cellular ROS production was measured in cells derived from a

primary GBM tumour, T1 and T2 using a DCFDA assay. Treatment with 5μM VP for 2 h caused a significant increase in ROS production which was suppressed upon co-treatment with 3 mM 4-Hydroxy-TEMPO (TPL; 2 h pre-treatment). **(b)** ROS production was quantified in U343 cells following treatment with vehicle (0.1% DMSO), 5 µM protoporphyrin IX (PPIX) or 5 µM PPIX + 3 mM TPL for 2 h in both 21% and 1% O_2. PPIX was unable to increase ROS production compared to vehicle control. **(c)** ROS levels were seen to significantly increase under hypoxia in U343 cells following 5 µM VP for 2 h compared to normoxic conditions. **(d)** Increasing concentrations of ferric chloride (Fe^{3+}; 3-500 µM) was added to 30 µM VP and the absorbance spectra were measured between 300-800 nm. A closer look around the VP activation point at 686 nm shows a progressive shift and reduction in absorbance with increasing concentrations of ferric chloride. **(e)** Other divalent ions were measured; ferrous chloride (Fe^{2+}) magnesium chloride ($MgCl_2$), and Zinc Chloride ($ZnCl_2$) which did not produce the same shift in spectra as ferric chloride. **(f**) Mass spectrometry analysis showed binding between verteporfin and Fe^{3+}, Fe^{2+} and Zn^{2+} but not Mg^{2+}. **(g)** First order binding kinetic graphs showing increased binding of Fe^{2+} and Zn^{2+} to VP but only after 8 h which is increased after 24 h. **(h)** 30 µM VP was added to 100 µM $FeCl_3$ which had been left at either 21% or 1% O_2 for 4 h and absorbance spectra were measured between 300-800 nm. First order binding kinetic graphs showed a similar binding of VP with $FeCl_3$ under normoxic and hypoxic conditions. It was noted that under hypoxia the VP maximal peak at 686 nm had shifted to 688 nm and so the binding ratio for hypoxia was calculated from the absorbance values at 672 nm and 688 nm. Experiments were conducted in at least biological triplicate with exception of the mass spectrometry data, which was run once. DCFDA experiments were conducted in a minimum of technical triplicate for each experiment. A one-way (Fig. S4a) and two-way (Fig S4b and c) ANOVA with Tukey post-hoc analysis was conducted. Data is presented as mean ± S.E.M, * $p<0.05$ ***$p<0.001$.

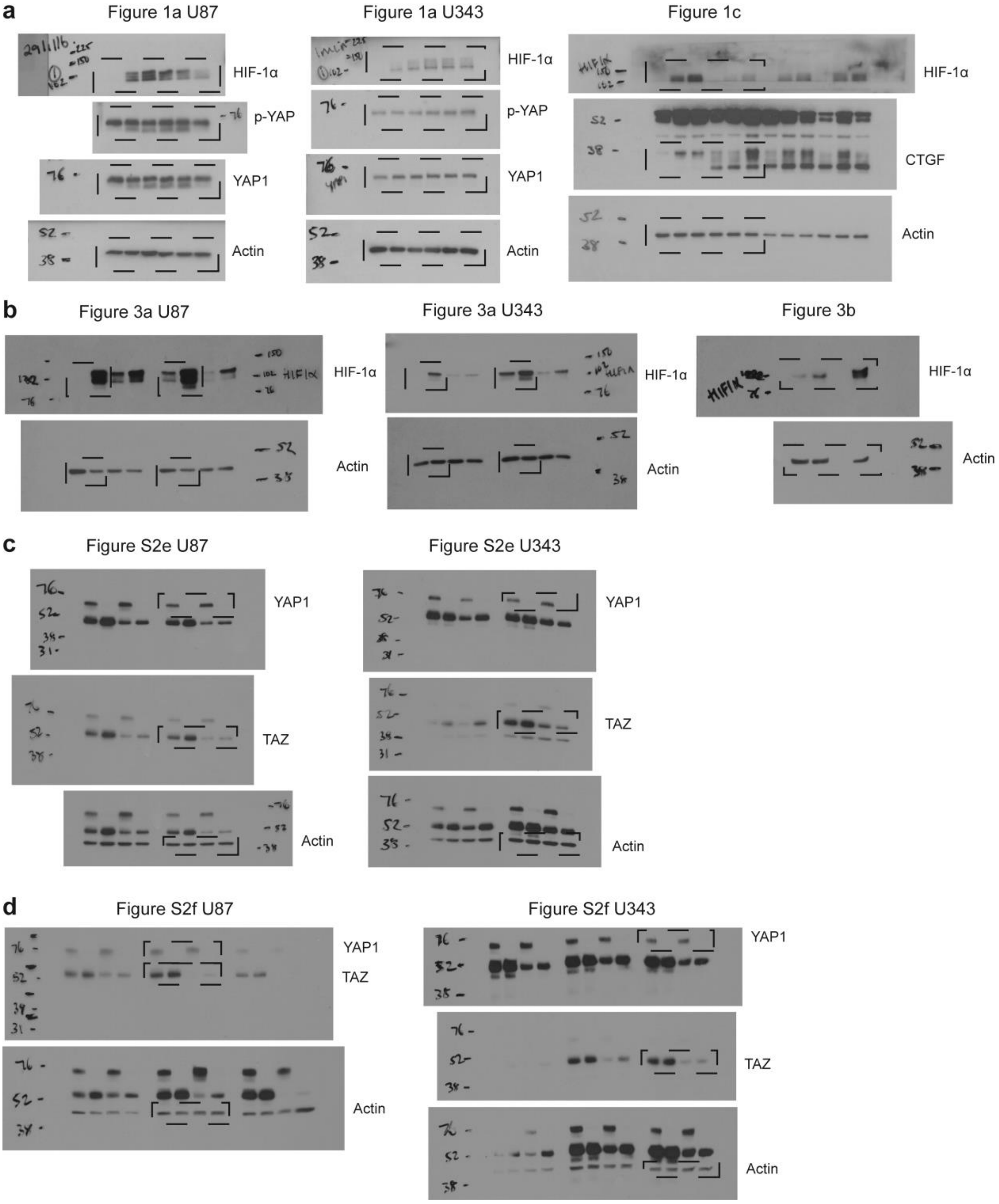

Supplementary Figure S5: Uncropped western blot images from the main paper and supplementary information. Blots are labelled with the corresponding figure. **(a)** Blots from figure 1. For Figure 1c, the band corresponding to CTGF is seen at 38 kDa. **(b)** Blots from figure 3. **(c)** Blots from supplementary figure S2e. **(d)** Blots from supplementary figure S2f. Dashed lines indicate the position where the blot was cropped.
